# Role of the Genetic Variants of Fetuin-A and Adiponectin in Type 2 Diabetes Mellitus: A Narrative Review

**DOI:** 10.7759/cureus.95914

**Published:** 2025-11-01

**Authors:** Huma Khan, Vishal Parmar, Mohammad Mustufa Khan, Saba Khan, Roshan Alam

**Affiliations:** 1 Department of Biochemistry, Integral Institute of Medical Sciences & Research (IIMSR), Lucknow, IND; 2 Department of Medicine, Integral Institute of Medical Sciences & Research (IIMSR), Lucknow, IND

**Keywords:** adiponectin, fetuin-a, genetic variants, metabolic regulation, t2dm

## Abstract

Type 2 diabetes mellitus (T2DM) is a global health concern driven by insulin resistance and beta-cell dysfunction. Understanding the genetic basis of T2DM is crucial for effective interventions. This narrative review explores the involvement of the genetic variants of fetuin-A and adiponectin, adipokines crucial for metabolic regulation, in T2DM risk. Fetuin-A, primarily produced by the liver, and adiponectin, secreted by the adipose tissue, play pivotal roles in the metabolism of glucose and the sensitivity of insulin. This review explores the genetic variants of fetuin-A and adiponectin in T2DM, highlighting their roles in metabolic regulation and insulin sensitivity. It discusses their association with T2DM onset, potential as diagnostic biomarkers, and therapeutic targets. The review underscores the importance of further research to understand these associations and their clinical implications for personalized treatment.

## Introduction and background

Insulin resistance and beta-cell dysfunction are widely acknowledged as major factors for promoting type 2 diabetes mellitus (T2DM) [1]. The worldwide burden of T2DM is a significant concern, with approximately 463 million adults affected worldwide in 2019, and this number is projected to escalate to 700 million by 2045 [2]. Understanding the genetic basis of T2DM is crucial for developing effective interventions to address this growing health crisis.

Recent research has brought to light that a family of proteins, which are secreted from the adipose tissue known as adipokines, have a major role in regulating glucose metabolism. Studies have provided insights into the mechanisms underlying glucose regulation across different age groups. Unlike adipokines derived from fat cells, fetuin-A, also referred to as α-Heremans-Schmid glycoprotein (AHSG), is released from the liver [3]. In vitro experiments have revealed that fetuin-A binds reversibly to the insulin receptor tyrosine kinase in peripheral tissues, thereby obstructing the insulin-induced intracellular signalling cascade, leading to peripheral insulin resistance [4-6]. Correspondingly, studies on fetuin-A knockout mice have demonstrated increased insulin sensitivity, whereas wild-type mice treated with exogenous fetuin-A exhibit acute insulin resistance [7].

Adiponectin, classified as an adipocytokine, is also involved in lipid and glucose metabolism, energy homeostasis, and inflammatory pathways [8-12]. Circulating concentrations of adiponectin are decreased in individuals with T2DM [13-16]. Low plasma adiponectin levels are also observed in rodent models [17], and mice lacking adiponectin exhibit diet-induced insulin resistance [18,19]. Adiponectin's insulin-sensitizing effects are believed to be mediated by increased fatty acid oxidation and glucose uptake [17,20] and the suppression of gluconeogenesis [21,22] through the AMP-activated protein kinase channel [23,24] and peroxisome proliferator-activated receptor gamma (PPAR-γ) [25]. This review explores the roles of adipokines like adiponectin and fetuin-A in insulin resistance and beta-cell dysfunction, pivotal factors for T2DM, offering insights for potential therapeutic targets and preventive strategies.

## Review

In the past decade, there has been a growing interest in investigating the genetic variants associated with T2DM. Genetic research has provided a better understanding of pathophysiology related to T2DM and has the potential to identify novel therapeutic targets [26,27]. In this article, we will explore the current evidence on the genetic variants of two biomarkers, fetuin-A and adiponectin, and their association with T2DM. We will discuss the findings from various studies and their implications for understanding the genetic basis of T2DM.

Methodology

A narrative review approach was employed to explore the association between genetic variants of fetuin-A and adiponectin with the development of T2DM. A comprehensive literature search was conducted across MEDLINE, PubMed, Google Scholar, CINAHL (Cumulative Index to Nursing and Allied Health Literature), and Scopus, covering studies published between January 1, 2000, and June 30, 2024. The year 2000 was selected as the starting point to capture research from the modern genetic era, when molecular and genomic techniques became more widely applied to metabolic disorders. The search strategy used key terms and Boolean combinations such as "fetuin-A", "AHSG gene", "adiponectin", "ADIPOQ polymorphism", "genetic variants", and "type 2 diabetes mellitus". Two researchers independently screened titles, abstracts, and full texts to determine relevance, resolving disagreements through discussion and consensus. In total, 203 potentially relevant studies were identified, and 96 full-text articles were reviewed in detail. Studies examining the relationship between fetuin-A or adiponectin gene polymorphisms and T2DM susceptibility or onset were included. The findings from the eligible studies were synthesized narratively to summarize key patterns, highlight knowledge gaps, and identify emerging trends in the genetic understanding of T2DM.

Overview of fetuin-A

**Figure 1:**
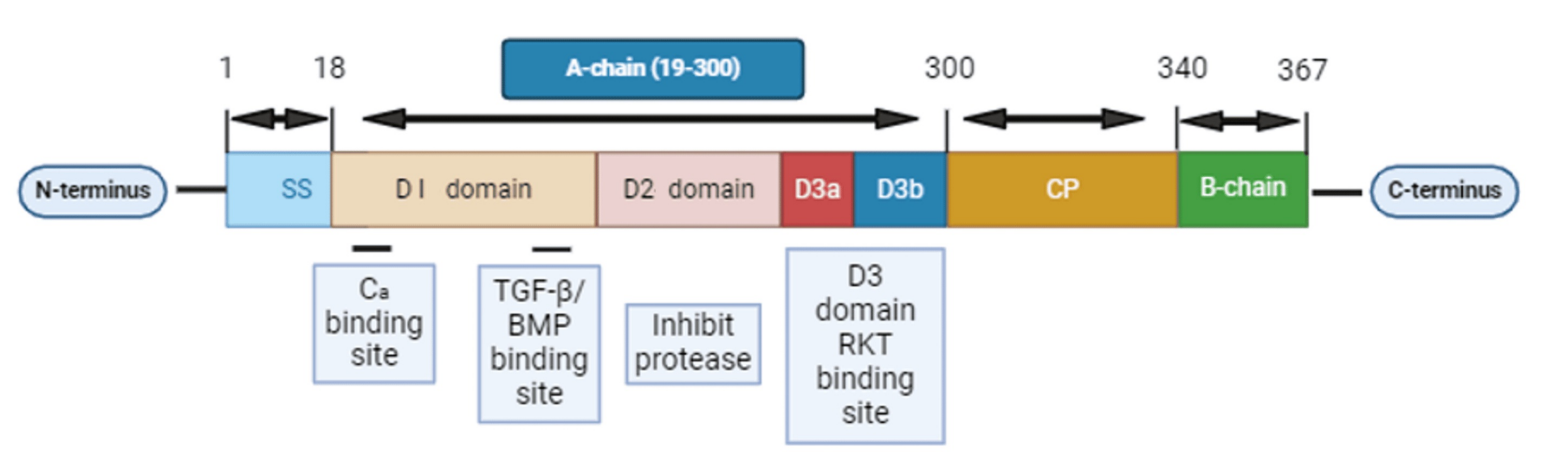
Structure of fetuin-A Fetuin-A is composed of two cystatin-like domains (D1 and D2) and a smaller C-terminal domain (D3), which is further divided into subdomains D3a and D3b. The SS facilitates protein secretion, while the CP links the cystatin domains. SS: signal sequence; CP: connecting peptide; D3a: domain 3a; D3b: domain 3b; RTK: receptor tyrosine kinase; TGF-β: transforming growth factor-beta; BMP: bone morphogenetic protein Image was created with BioRender.com

Fetuin-A, also recognized as AHSG, is a hepatic protein that serves as a systemic inhibitor of ectopic calcification [28]. It has also been noted for its insulin signalling ability [3,29]. The structure of fetuin-A is shown in Figure 1.

Several prospective population-based studies have investigated the relationship between circulating fetuin-A levels and the risk of cardiovascular disease (CVD) and/or T2DM, producing conflicting results [30-33]. Few studies reported that elevated plasma fetuin-A levels were associated with increased risks of myocardial infarction, ischemic stroke, and T2DM [31,32]. Conversely, a study by Rancho Bernardo found that higher levels of fetuin-A were linked to a reduced risk of mortality with CVD and an increased mortality in CVD with T2DM [32]. Similar trends were observed in another study, "Cardio-vascular Health Study", where elevated fetuin-A levels were linked with decreased CVD risk in non-diabetic individuals but exhibited an opposite trend in those with T2DM [33]. A Mendelian randomization analysis did not find any evidence for the relationship between fetuin-A and T2DM [34] as the publication of these earlier Mendelian randomization studies and new genetic variants associated with fetuin-A has been identified through genome-wide association studies [35]. Furthermore, advancements in Mendelian randomization methodologies have enhanced the detection and consideration of potential biases. Additionally, the emergence of large biobanks, integrating diverse phenotypic and genetic datasets, enables the assessment of the comorbidity with fetuin-A.

Genetic Variants of Fetuin-A

Several genetic variants have been associated with AHSG (fetuin-A) gene regulation and circulating protein levels. Notable single-nucleotide polymorphisms (SNPs) include rs4615068, rs6809265, rs2070633, rs2248690, and rs2070635, which have been implicated in modulating AHSG gene expression and serum fetuin-A concentrations. Among these, rs4917, one of the most extensively studied variants, has been shown to significantly influence circulating fetuin-A levels, with minor alleles generally linked to reduced protein concentrations in both European and African American populations [33,35]. Similarly, rs2070633 and rs2248690 were identified by Kröger et al. [34] as key variants affecting transcriptional activity within the AHSG promoter region, thereby altering fetuin-A expression. These SNPs have subsequently been used in Mendelian randomization studies to investigate the potential causal effects of fetuin-A on cardiometabolic outcomes, including CVD and T2DM.

Correlations of Fetuin-A

An association between genetically predicted fetuin-A and T2DM, with discrepancies possibly attributed to stronger genetic instruments and variant exclusions [34-36]. Previous experiments in adipocytes from mice and humans have demonstrated that fetuin-A is essential for inducing an inflammatory signalling pathway leading to insulin resistance [37]. Stefan and Häring's observational study of 347 participants at high risk of CVD disease highlighted an interaction between fetuin-A and insulin sensitivity [38,39].

Fetuin-A increases coronary artery disease risk in individuals having T2DM but not in those without it [39,40], and a decreased risk of myocardial infarction is observed in women but not in men, highlighting potential gender-specific alterations in the pathophysiological role of fetuin-A in acute coronary syndrome [39,40]. In line with prior studies, the association between genetically predicted fetuin-A and ischemic stroke was not found [36,39].

Overview of adiponectin

**Figure 2:**
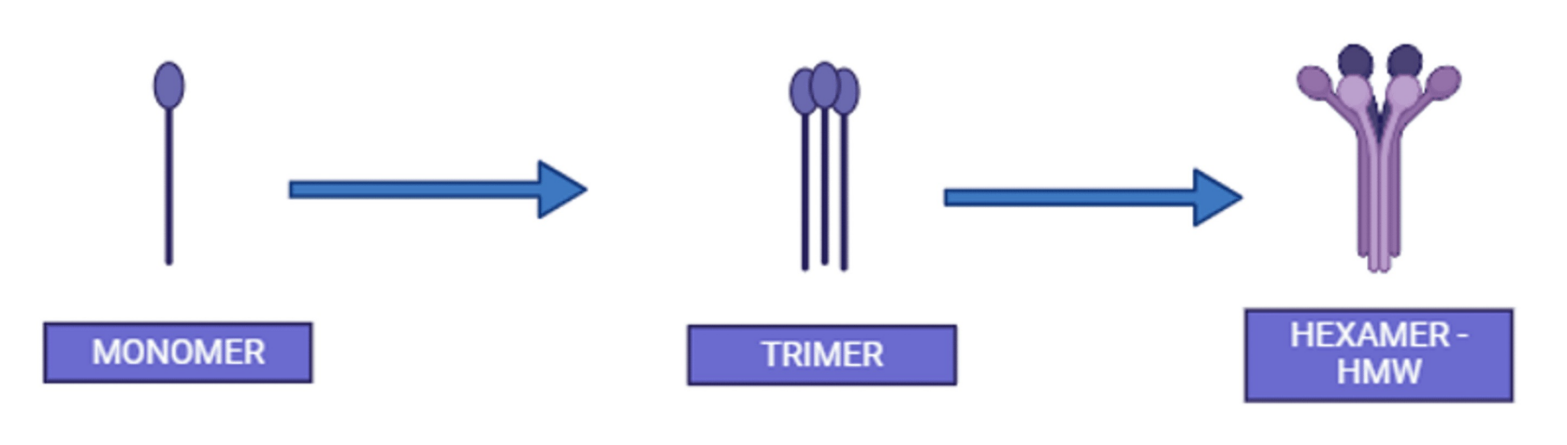
Structure of adiponectin HMW: high-molecular weight Image was created with BioRender.com

Adiponectin has been independently recognized by various research groups [8,41-43]. It serves as an adipokine and is renowned for having multifaceted benefits, including anti-diabetic, anti-inflammatory, cardioprotective, and anti-atherogenic roles [14,44,45]. In human circulation, adiponectin is present at concentrations ranging from approximately 5 to 30 μg/mL and consists of about 244 amino acids [44-46]. In obese individuals, pigs, and rodents, the expression of adiponectin and total serum levels exhibits a decline [46,47]. Notably, there exists a sexual dimorphism in adiponectin expression, with men demonstrating lower levels compared to women [46]. Adiponectin has a molecular weight of 28-30 kDa, which further forms homo-oligomers including the low-molecular-weight trimeric form, medium-molecular-weight hexameric form, and high-molecular-weight form [43,48]. The structure of adiponectin is shown in Figure 2.

Genetic Variants of Adiponectin

Adiponectin, encoded by the APM1 gene, spans three exons over 16 kb and is situated on chromosome 3's long arm in the region 3q27, closely linked to quantitative trait loci (QTL) for metabolic syndrome (MetS) and T2DM [49]. Specific SNPs and mutations in the APM1 gene are associated significantly with T2DM and hypoadiponectinemia, although studies on SNPs have yielded contradictory results [20,50-55].

Various cross-sectional investigations have identified associations between SNPs in the adiponectin gene and diabetes risk. These SNPs are instrumental in evaluating common variant associations with adiponectin levels and susceptibility to T2DM. Notably, SNPs such as rs1501299, rs2241766, rs266729, rs17366743, rs17300539, rs182052, rs822396, rs17846866, rs3774261, and rs822393 have been shown to play significant roles in T2DM pathogenesis. Furthermore, rs2241766 and rs266729 exhibit substantial associations with gestational diabetes mellitus, suggesting that certain adiponectin variants may influence glucose metabolism across different metabolic states. Collectively, these findings underscore the pivotal role of adiponectin gene polymorphisms in modulating diabetes susceptibility, particularly in T2DM, the predominant form of the disease [56].

The monomeric structure of adiponectin comprises four distinct regions: (1) amino-terminal peptide, (2) a short hyper-variable region, (3) a collagen-like domain with 22 Gly-X-Pro or Gly-X-Y repeats, and (4) a carboxy-terminal globular domain C1q-like [43]. In serum, adiponectin exists in two primary forms: the full-length form (fAdiponectin) and the globular form (gAdiponectin) [56,57]. Proteolytic cleavage of fAdiponectin produces gAdiponectin, lacking the collagen-like domain, which enables trimer formation but not high-molecular-weight oligomerization [58,59]. Recent findings indicate that the monomeric form stimulates AMP-activated protein kinase (AMPK) activation, enhancing fatty acid oxidation and peripheral glucose uptake [60]. Moreover, elevated gAdiponectin levels improve the energy metabolism of the whole body as well as adipose tissue functionality [47,61,62]. Posttranslational modifications, including hydroxylation, glycosylation of lysine residues, and proline hydroxylation, are crucial for adiponectin multimer assembly, primarily mediated by cysteine for disulfide bond formation [63]. Adiponectin oligomers play a role in its biological activities, with high-molecular-weight oligomers being particularly pertinent to the sensitivity of insulin, increasing the risk for obesity [64]. Consequently, the high-molecular-weight/total adiponectin ratio is deemed more informative than total adiponectin alone for assessing the risk of conditions like obesity, insulin resistance, T2DM, MetS, and CVDs [65-67]. Obesity, assessed through indicators like waist circumference (WC) and body mass index (BMI), is a well-established risk factor for T2DM [68].

Recent research suggests that WC, BMI, and adiponectin are significant predictors of diabetes incidence, with pooled relative risks per standard deviation indicating similar associations for all three indicators [68]. Adiponectin, secreted exclusively by the adipose tissue, plays a vital role in both lipid and glucose metabolism, circulating abundantly in the plasma [69-73]. It diminishes insulin resistance by enhancing lipid oxidation in various organs, including the muscle, pancreas, and liver [17]. Plasma adiponectin concentrations exhibit sex-dependent differences, with higher levels among women and reductions observed in individuals with obesity, diabetes mellitus, or coronary heart disease [16,74]. While numerous studies have examined the independent associations of WC, BMI, and adiponectin with diabetes risk, investigations into their combined effects remain limited in the existing literature.

Correlations of Adiponectin

Adiponectin is released exclusively by adipose cells, while fetuin-A is primarily produced by hepatocytes; however, emerging evidence suggests that the adipose tissue can also synthesize and secrete fetuin-A [43,75,76]. Given that adiponectin and fetuin-A exert opposing effects on metabolic regulation, their ratio may serve as a useful biomarker for evaluating metabolic disorders such as T2DM. Genetic variations in the adiponectin gene can influence circulating adiponectin levels and, consequently, alter T2DM susceptibility. In a study by Gui et al., the rs1501299 SNP of the adiponectin gene was analyzed in a Chinese population [77]. The results demonstrated that individuals with the CC genotype had significantly higher adiponectin levels and a lower risk of developing diabetes compared to those with the TT genotype.

Furthermore, a meta-analysis by Wu et al. investigated the association between the rs2241766 SNP and T2DM risk [78]. The meta-analysis included 16 studies and found that the T allele of the rs2241766 SNP was linked with a greater risk of T2DM. These findings highlight the importance of genetic variants of the gene associated with adiponectin.

Interaction between fetuin-A and adiponectin

Adiponectin and fetuin-A go against one another, even though they are closely linked to T2DM and cardiovascular disorders. Previous genome-wide scans offered strong evidence that the genes for fetuin-A and adiponectin were found on chromosome 3q27-qter, a region linked to a hereditary vulnerability to early-onset diabetes. Therefore, it is believed that fetuin-A and adiponectin work together in T2DM-related disorders and that the fetuin-A/adiponectin (F/A) ratio is a better indicator of the onset and course of these disorders than either protein by itself [77-79]. Emerging evidence suggests an intricate interplay between T2DM and fetuin-A. In a study by Zhou et al., the F/A ratio was investigated as a more sensitive indicator for evaluating MetS [79].

Mechanisms of action

Fetuin-A inhibits insulin receptor tyrosine kinase activity, thereby promoting insulin resistance. It has also been implicated in inflammatory responses and lipid metabolism dysregulation. In contrast, adiponectin exerts insulin-sensitizing and anti-inflammatory effects by enhancing insulin signaling pathways, improving glucose uptake, and reducing inflammation in the adipose tissue and liver. Earlier studies identified fetuin-A primarily as a systemic factor maintaining the solubility of calcium and phosphorus in serum, thus preventing vascular calcification [80]. However, subsequent research demonstrated that elevated circulating fetuin-A levels correlate positively with subclinical inflammation in large population-based studies, suggesting its potential involvement in metabolic dysfunction [81]. Although higher fetuin-A levels have not been consistently linked to diabetes risk among individuals with normal glucose tolerance, a positive association has been observed in those with elevated plasma glucose levels within the non-diabetic range. Importantly, while increased fetuin-A levels are often accompanied by reduced adiponectin concentrations, this relationship is associative rather than directly causal, reflecting their opposing roles in metabolic regulation.

The most prevalent protein derived from white adipose tissue is adiponectin. Tissues that include the liver, kidney, pancreas, skeletal muscle, bone, and glands express it [82]. Adiponectin exhibits anti-diabetic, anti-oxidative, and anti-inflammatory properties that contribute to maintaining lipid homeostasis and reducing insulin resistance, oxidative stress, and inflammation associated with T2DM and CVD [23]. In T2DM mice, gluconeogenic enzyme expression is inhibited by the high amounts of adiponectin in circulation, low levels of adiponectin linked to obesity-related insulin resistance, and MetS [83]. Adiponectin stimulates protein kinase (AMPK), which in turn is activated by adenosine monophosphates [84]. This leads to the enhanced oxidation of fatty acids and blood sugar utilization.

Adiponectin levels have been proposed by Jiang et al. as a strong predictor of incident prediabetes and the development of T2DM [85]. However, Banerjee et al. found that decreased adiponectin concentrations were associated with an increased risk for the development of T2DM and prediabetes, which may be useful in the identification of biomarkers and the creation of experimental models [86]. Mutations in the ADIPOQ gene are doing the multimerization process of proteins that can affect the activity of proteins biologically by disturbing the assembling of trimeric and high-molecular-weight (multimeric) forms of proteins that may lead to hypoadiponectinemia [87]. Gene variants of ADIPOQ +10211T/G (rs17846866) and +276G/T (rs1501299) were examined in the Gujarat population and found to be significantly associated with T2DM. That the -11377C/G (rs266729) gene variant of the ADIPOQ gene was associated with T2DM in the population of Jammu and Kashmir and both were associated with hypoadiponectinemia were also investigated [88]. Adiponectin via its receptors, i.e., AdipoR1 and AdipoR2, through AMP-activated protein kinase activity regulates carbohydrate, cholesterol, and fatty acid metabolism [89].

Clinical implications

The cumulative findings regarding the impact of genetic variants associated with fetuin-A and adiponectin on T2DM carry substantial clinical implications. Primarily, these genetic markers present promising biomarkers for evaluating various risk factors for T2DM. Individuals harboring specific genetic variants may exhibit an elevated or diminished susceptibility to T2DM, paving the way for targeted preventive strategies. Additionally, comprehending the underlying mechanisms linking these genetic variants to T2DM can guide the development of innovative therapeutic approaches. Targeting the pathways influenced by fetuin-A and adiponectin holds potential for novel interventions related to the treatment of T2DM. Recent research indicates that interventions such as PPAR-γ agonists like pioglitazone, gastric bypass surgery, and lifestyle modifications involving diet and physical activity can significantly reduce fetuin-A levels, offering multifaceted avenues for managing T2DM [90-92]. Notably, these same interventions have been shown to increase adiponectin concentrations, thereby improving insulin sensitivity and enhancing metabolic balance. Thus, by simultaneously lowering fetuin-A and elevating adiponectin, such therapeutic strategies may favorably modulate the F/A ratio, which serves as an emerging biomarker of metabolic health and glycemic control [90-92].

## Conclusions

The collective evidence suggests that genetic variants in the fetuin-A and adiponectin genes develop T2DM. The T nucleotide of the rs4917 SNP in the fetuin-A gene and the CC genotype of the rs1501299 SNP of the adiponectin gene may confer a protective effect against T2DM. The hypothesis that fetuin-A may contribute to the development of T2DM is supported by the finding that its levels can predict the incidence of the disease independent of other known risk factors. Furthermore, the F/A ratio has emerged as a potentially valuable marker for evaluating T2DM risk.
